# Fish oil supplementation attenuates cognitive impairment by inhibiting neuroinflammation in STZ-induced diabetic rats

**DOI:** 10.18632/aging.103426

**Published:** 2020-08-04

**Authors:** Gengyin Wang, Xiaohan Zhang, Xunyi Lu, Jiang Liu, Zhiyong Zhang, Zifeng Wei, Zeteng Wu, Jinhua Wang

**Affiliations:** 1 School of Basic Medicine, North China University of Science and Technology, Tangshan 063210, Hebei Province, PR China; 2 Department of Neurology, Taizhou Central Hospital (Taizhou University Hospital), Taizhou 318000, Zhejiang Province, PR China; 3 The Neurology Institute of Taizhou University, Taizhou 318000 Zhejiang Province, PR China

**Keywords:** fish oil, diabetic cognitive impairment, neuroinflatmmation, oxidative stress, hippocampus

## Abstract

Type 2 diabetes mellitus (T2DM) markedly impairs human health. During T2DM development, some patients experience cognitive dysfunction and behavioral deficits, which are characterized by neuronal injury and memory loss. It has been reported that the incidence of dementia in middle-aged and elderly patients with diabetes is significantly higher than that in normal elderly patients. Currently, the pathogenesis of cognitive dysfunction in diabetes remains unknown, and there is no standard or specific method to diagnose the disease in clinical practice. Evidence has shown that fish oil (FO) can alleviate depressive-like behaviors by attenuating neuroinflammation in a rat model, and improve cognitive dysfunction by inhibiting apoptosis. Therefore, it is reasonable to speculate that FO may reduce cognitive impairment by attenuating neuroinflammation in diabetic rats. In the present study, we investigated the effects of FO supplementation on cognitive dysfunction in a streptozotocin-induced diabetic rat model. FO administration for 10 weeks improved spatial learning and memory as evaluated by performance in the Morris water maze (MWM). Besides, FO significantly improved the morphology of neurons in the hippocampus and cortex of diabetic rats and reduced the neuronal nuclear condensation. Moreover, FO decreased the mRNA expression of IL-1β, IL -6, and TNF-α and increased the mRNA expression of IL-4 and IL-10 in the cortex and hippocampus. FO also attenuated the brain inflammatory cascade and simultaneously reduced diabetes-induced oxidative stress. In addition, FO increased the protein expression of Nrf2 and HO-1 in cortex and hippocampus of diabetic rats. These results provide a novel horizon for the study of neuroprotective effect of FO and further clarify the connections among inflammation, oxidative stress and diabetes-induced cognitive impairment.

## INTRODUCTION

Type 2 diabetes mellitus (T2DM) is a common disease among elderly individuals, is highly prevalent worldwide, and is closely related to cognitive impairment. A large amount of epidemiological data has shown that the risk for cognitive impairment is doubled in individuals with T2DM, and cognitive impairment may be related to worse T2DM outcomes [1, 2]. However, the mechanism through which T2DM diabetes induces cognitive dysfunction and eventually dementia is still unknown. Much research regarding the pathogenesis of cognitive dysfunction in T2DM has been performed. Imaging studies have found structural abnormalities in the brains of patients with diabetes, which may accelerate cognitive decline [3, 4]. In rat model experiments, the number of hippocampal neurons obviously decreased, and abnormal hippocampal neuron was observed by optical microscopy [5]. Some studies have demonstrated that neuroinflammation and oxidative stress may be the key contributors to the development of diabetes-related cognitive dysfunction [6, 7]. After these risk factors are activated, the cognitive function of patients with diabetes declines, eventually leading to AD, which markedly affects the life of patients, and negatively impacts on patients' families and public health. Unfortunately, there is no effective drug that can prevent or slow the progression of cognitive impairment in diabetes. Therefore, developing effective drugs to prevent or delay cognitive impairment in diabetes remains a challenge.

Fish oil is a natural health product refined from the fat of marine fish and is rich in samarium compounds and monounsaturated fatty acids. Marine fish oil contains four long carbon chain polyunsaturated fatty acids, including arachidonic acid (AA), eicosapentaenoic acid (EPA), docosapentaenoic acid (DPA) and docosahexaenoic acid (DHA). However, in general, among the four fatty acids, EPA and DHA are present at the highest levels in FO, and are also beneficial to human health. FO has the effect of enhancing immunity [8], improves rheumatoid arthritis [9], and fatty liver [10] and inhibits cancer [11]. Recent studies also suggested that FO could be a potential drug to prevent and treat diabetes [12] and inhibit middle-aged dementia [13]. To understand how fish oil can prevent diabetes-associated cognitive impairment, we established an animal model.

## RESULTS

### FO suppressed diabetes-induced behavioral deficit

**Figure 1:**
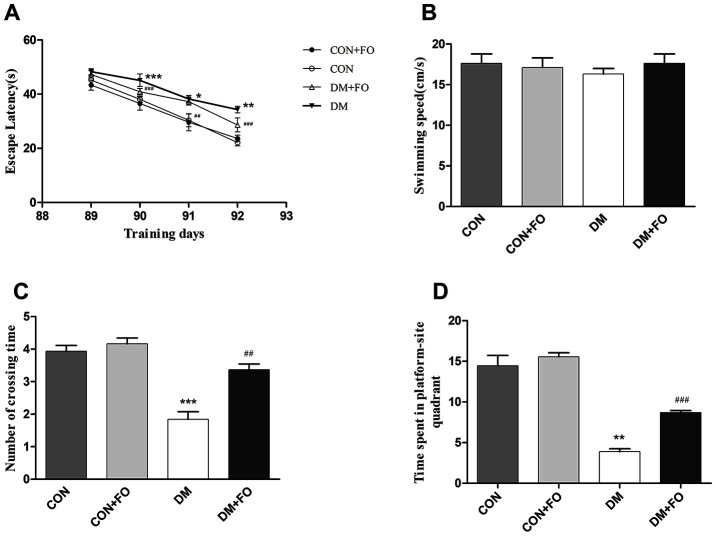
**The spatial learning and memory ability of rats in different groups.** (**A**) Escape latency in navigation training. (**B**) Swimming speed in the probe test. (**C**) The number of platform quadrant crossings. (**D**) Time spent in the target quadrant. Data were analyzed using repeated-measures ANOVA with *post hoc* Bonferroni tests. Data are shown as the mean ± SEM (n=7). ^*^ *P<*0.05, ^* *^P<0.01 and ^***^P<0.001 vs. the CON group; *^#^ P<*0.05, *^##^P<*0.01 and *^###^P<*0.001 vs. the DM group.

The MWM test was carried out to assess whether FO improved spatial learning and memory ability in DM rats. During the five day test, there was no obvious difference in swimming speed between the groups (Figure 1B). In the orientation navigation test, rats in the DM group spent more time finding the hidden platform than the rats in the CON group on day 90, 91 and 92 (Figure 1A); FO administration reduced the escape latency of DM rats on day 90, 91 and 92 (Figure 1A). In the probe test, as shown in Figure 1C, the number of crossings in DM group was much less than that in the CON group; after FO treatment, we observed a significant improvement in the DM+FO group. The DM group, spent significantly less time in the target quadrant than the CON group; after FO treatment, rats spent markedly more time in the target quadrant than did rats in the DM group (Figure 1D).

### FO changed morphology of the cortex and hippocampus

**Figure 2:**
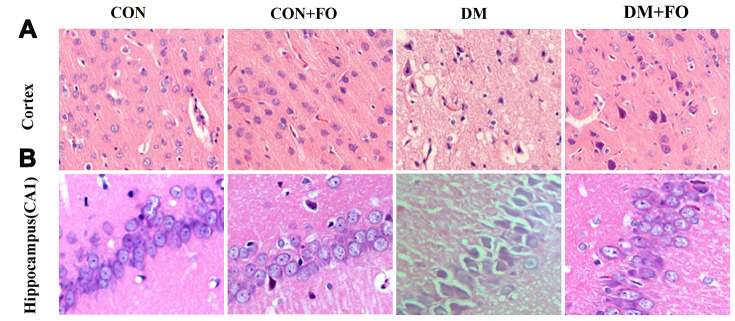
**Morphological changes in cortical and hippocampal neurons.** (**A**) Morphological changes in cortical nerve cells in rats from different groups, CON, CON+FO, DM and DM+FO; (**B**) Morphological changes of hippocampal neurons in rats from different groups, CON, CON+FO, DM and DM+FO. (n=8).

H&E staining was used to observe the change in morphology in the cortex and hippocampus under an optical microscope. Few abnormal neurons were observed in the CON and CON+FO groups. In the DM group, the neurons were abnormally and disorderly arranged, the staining was clouded and the number of neurons was decreased. FO treatment alleviated the abovementioned abnormal morphological changes (Figure 2).

### Effects of FO on neuroinflammation

**Figure 3:**
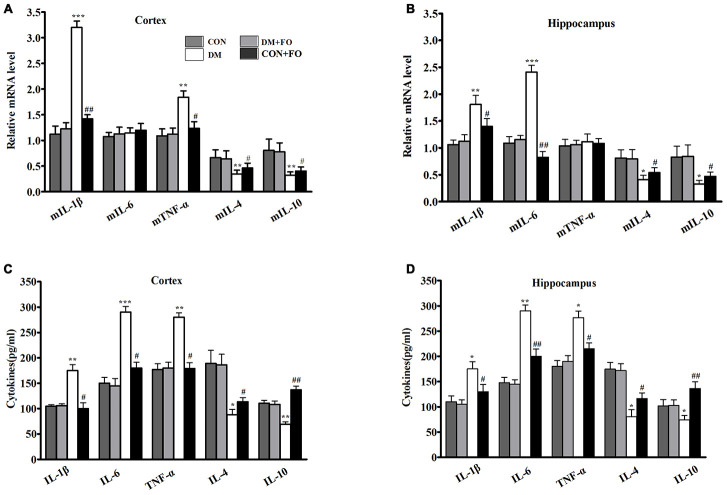
**Proinflammatory cytokine in different groups.** (**A**) Expression of mIL-1β, mIL-6, mTNF-α, mIL-4 and mIL-10 in the cortex. (**B**) Expression of mIL-1β, mIL-6, mTNF-α, mIL-4 and mIL-10 in the hippocampus. (**C**) Content of IL-1β, IL-6, TNF-α, IL-4 and IL-10in the cortex. (**D**) Content of IL-1β, IL-6, TNF-α, IL-4 and IL-10 in the hippocampus. Data are shown as the mean ± SEM (n=7) ^*^P<0.05, ^**^P<0.01 and ^***^P<0.001 vs. the CON group; *^#^ P<*0.05, *^##^P<*0.01 and *^###^P<*0.001 vs. the DM group.

We used an ELISA kit to determine whether DM could stimulate a neuroinflammatory response. As hypothesized, in the brain region of the cortex, the levels of IL-1β, IL-6, and TNF-α were much higher in the DM group than in the CON group, while the levels of IL-4 and IL-10 were lower in the DM group than in the CON group (Figure 3C). FO supplementation suppressed pro-inflammatory cytokine levels (IL-1β, IL-6, and TNF-α) and enhanced anti-inflammatory cytokine levels (IL-4 and IL-10) in the cortex (Figure 3C). In the hippocampal region, IL-1β, IL-6, and TNF-α levels were increased, and IL-4 and IL-10 levels were decreased in the DM group compared with the CON group (Figure 3D). FO supplementation inhibited the increase in pro-inflammatory cytokines (IL-1β, IL-6, and TNF-α) and the decrease in anti-inflammatory cytokines (IL-4 and IL-10) in hippocampus (Figure 3D).

To further understand the effect of fish oil on inflammatory cytokines, we measured the mRNA expression of the five inflammatory cytokines in cortex and hippocampus, using RT-PCR. The findings showed that the transcription levels of the pro-inflammatory cytokines IL-1β and TNF-α were obviously increased, and IL-4 and IL-10 were significantly decreased in the cortex (Figure 3A), whereas FO administration markedly inhibited the diabetes-induced upregulation of IL-1β and TNF-α and downregulation of IL-4 and IL-10 (Figure 3A). Rats in the DM group had clearly higher expression of IL-1β and TNF-α and lower expression of IL-4 and IL-10 than rats in the CON group. The rats that received FO had obviously lower expression of IL-1β and TNF-α and higher expression of IL-4 and IL-10 than the rats in the DM group (Figure 3B). However, the mean expression of IL-6 in the cortex and the mean expression of TNF-α in the hippocampus were not significantly different among the groups.

### Effects of FO on oxidative stress

**Figure 4:**
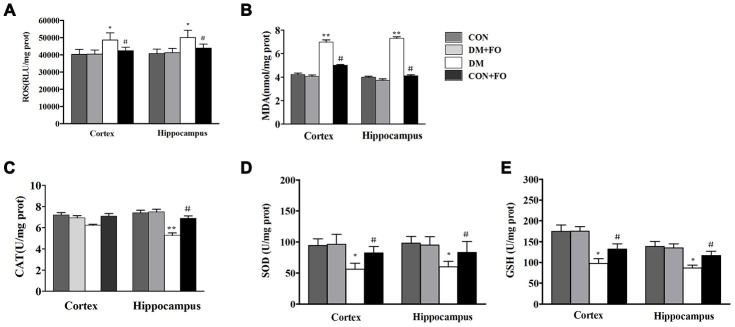
**Oxidative stress measurements in different groups.** (**A**) ROS levels in the cortex and hippocampus. (**B**) MDA level in the cortex and hippocampus. (**C**) CAT activities in the cortex and hippocampus. (**D**) SOD activities in the cortex and hippocampus. (**E**) GSH levels in the cortex and hippocampus. Data are shown as the mean ± SEM (n=7) ^*^P<0.05, ^* *^P<0.01 and ^* * *^P<0.001 vs. the CON group; *^#^ P<*0.05, *^##^P<*0.01 and *^###^P<*0.001 vs. the DM group.

To identify the mechanisms underlying the neuroprotective effect of FO, we also measured the levels of ROS and oxidative stress markers including MDA, CAT, SOD and GSH in the cortex and hippocampus. ROS and MDA were dramatically increased, and SOD and GSH were dramatically decreased in the cortex and hippocampus of rats in the DM group compared to the CON group (Figure 4A, 4B, 4D, 4E), CAT was only observably decreased in the hippocampus of rats in the DM group compared with that of the CON group (Figure 4C). Treatment with FO reduced ROS and MDA levels both in the cortex and hippocampus (Figure 4A, 4B), increased the GSH level in the cortex and hippocampus (Figure 4E) and enhanced CAT in hippocampus and SOD activities (Figure 4C, 4D) in the cortex and hippocampus of the rats in DM group.

### Effects of FO on protein expression of Nrf2 and HO-1

**Figure 5:**
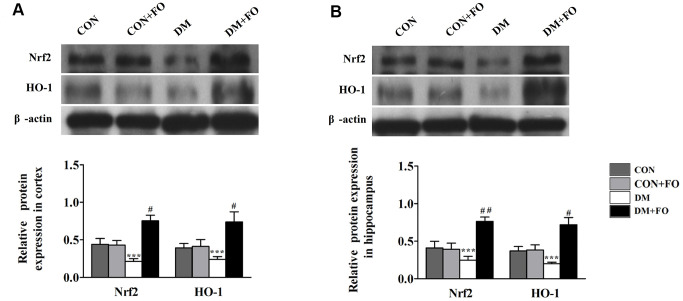
**Effects of FO on the expression of Nrf2 and HO-1.** (**A**) Expression of Nrf2 and HO-1 in the cortex. (**B**) Expression of Nrf2 and HO-1 in the hippocampus. Data are shown as the mean ± SEM (n=7) ^*^ *P<*0.05, ^* *^P<0.01 and ^* * *^P<0.001 vs. the CON group; *^#^ P<*0.05, *^##^P<*0.01 and *^###^P<* 0.001 vs. the DM group.

To further reveal the mechanism underlying the neuroprotective effect of FO, Nrf2 and HO-1 protein expression were measured by Western blot analysis. As shown in Figure 5, the protein expression of Nrf2 and HO-1 was decreased in the rats with diabetes compared to the rats in the CON group. FO treatment increased the protein expression of Nrf2 and HO-1 in both cortex and hippocampus.

## DISCUSSION

T2DM is an age-related metabolic disorder that is prevalent worldwide. The onset of diabetes generally occurs after the age of 35-40 [14]. As age and hyperglycemia duration increase, blood glucose worsens, and diabetic complications increase. Increasing evidence shows that T2DM is associated with cognitive impairment and dementia, and many epidemiological studies have shown that patients with T2DM have a significantly higher risk of developing AD [1, 15]. The mechanism by which T2DM causes neurodegenerative diseases remains unclear and no drugs effectively prevent or delay disease progression. Therefore, finding effective treatments or developing new drugs to fight diabetes cognitive impairment is an important challenge.

The results of this study provide empirical support that FO plays a significant role in improving cognitive function in diabetic rats. More specifically, FO improved behavioral abnormalities in diabetic rats and restored neuron morphology in the cortex and hippocampus which are the brain regions responsible for learning and memory. Moreover, our findings also demonstrated that the behavioral benefits provided by FO were involved in attenuating inflammatory cytokine expression and oxidative stress.

Our study showed that persistent hyperglycemia in rats could lead to behavioral abnormalities; however, FO administration could reverse the spatial learning and memory impairment induced by diabetes. The results were similar to those of a previous study [16], which reported that FO supplementation appears to improve memory and learning ability by inhibiting neuronal apoptosis in STZ- induced diabetic rats.

Proinflammatory cytokines, such as IL-6, IL-1β and TNF-α, are known for their role in neuronal death/loss. These proinflammatory cytokines have been hypothesized to induce neuroinflammation and then lead to oxidative stress by inducing ROS production and lowering antioxidant levels [17, 18], for example, SOD and GSH. Consistent with previous findings, our study demonstrated that STZ injection increased the expression of proinflammatory cytokines (IL-6, IL-1β and TNF-α) and the levels of ROS and MDA, whereas it decreased the expression of anti-inflammatory cytokines (IL-4 and IL-10) and the activity of CAT, SOD and GSH in the cortex and hippocampus. It was reported that intensive immunogenic responses and oxidative stress could cause neuronal damage [19]. H&E staining showed that a large number of neurons in the cortex and hippocampus died and the expression levels of pro-inflammatory cytokines were significantly increased. Based on inflammatory cytokine and oxidative stress marker results, we hypothesize that neuronal damage in diabetic rats is closely related to inflammatory cytokines and oxidative stress. However, FO alleviated the brain damage induced by diabetes. A large amount of evidence has shown that FO can reduce the expression of pro-inflammatory factors and thus suppress the immunogenic response in the liver and other tissues [20, 21]. Based on the above evidence, we measured the expression of inflammatory cytokines, and we found significantly reduced levels of ROS, IL-6, IL-1β and TNF-α and enhanced activities of CAT, SOD and GSH in the cortex and hippocampus. Previous studies have shown that the ω-3 fatty acid docosahexaenoic acid (DHA), one of the active ingredients in FO, is able to reduce the risk of AD and to ameliorate symptoms and enhance learning function by inhibiting the apoptosis of hippocampal neurons in STZ- induced diabetic rats [22, 23]. Therefore, we preliminarily showed that FO achieves neuroprotective effects by inhibiting the expression of pro-inflammatory factors and thereby inhibiting the immunogenic response and oxidative stress.

Recent studies have found that the Nrf2-ARE signaling pathway is a key pathway for cellular antioxidative stress. The antioxidant enzymes and phase II detoxifying enzymes regulated by this signaling pathway can remove harmful substances such as ROS and thus promote detoxification and neutralization [24]. When the Nrf2-ARE signaling pathway is activated, it can induce the transcription of a large number of protective genes such as HO-1, GST and NQO1, and then promote resistance to various oxidative stress induced damage to the body [25]. In addition to the antioxidative action of Nrf2, Nrf2 also affects the stimulation of cells and tissues by proinflammatory factors and reduces cell damage. Excessive inflammatory factors can further stimulate cells to produce ROS, and the activation of the Nrf2-ARE pathway can inhibit the mutual promotion of inflammatory factors and ROS. Therefore, we used western blotting to measure the expression of Nrf2 and HO-1, and verified the previous conclusions that the Nrf2-ARE pathway was activated by FO. In summary, we believe that FO inhibits inflammation and oxidative stress injury by activating the Nrf2-ARE pathway

## CONCLUSIONS

FO supplementation improved cognitive dysfunction in STZ-induced diabetic rats by inhibiting proinflammatory cytokine expression and oxidative stress by activating the Nrf2-ARE pathway Theclarification of these effects will contribute to the elucidation of the complex mechanisms of cognitive dysfunction in diabetes and serve as a basis for developing effective drugs that improve cognitive function in age-related dementia. Because the rats in this study had mild cognitive impairment similar to the early stages of AD in human patients, our findings may provide theoretical support for the study of potential diabetic cognitive impairment. Further research is needed to better understand the potential protective effect of FO against diabetes-induced cognitive decline.

## MATERIALS AND METHODS

Sixty experimental adult male Sprague-Dawley (SD) rats (160-180g) were provided by the Experimental Animal Center of North China University of Science and Technology. Animals were housed at a controlled temperature (22 ± 2°C) and under a 12:12 h light/dark cycle. All rats were treated in accordance with the Care and Use of Laboratory Animal guidelines and the protocols were approved by the Animal Ethics Welfare Committee of North China University of Science and Technology.

### Treatment

**Figure 6:**
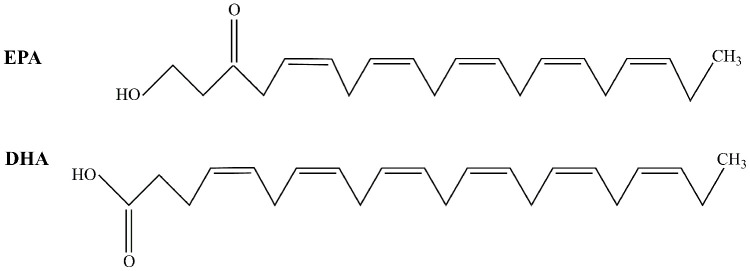
**The structure of EPA and DHA.**

**Figure 7:**
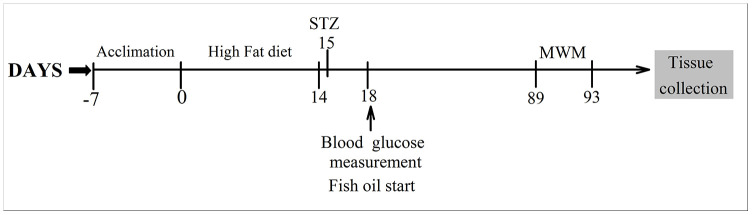
**Timeline of the experimental procedures.**

Diabetes was established with a high-fat diet (10% cholesterol 10 ml/kg) for two weeks. After overnight fasting, rats were injected intraperitoneally with 40 mg/kg streptozotocin (STZ, Sigma, USA) [26]. Control rats received a citrate buffer injection. Before high-fat diet consumption, all the animals were divided into four groups. The animals in the control group (CON group, n=15) and diabetes mellitus group (DM group, n=15) were fed standard rat feed. The animals in the control +FO group (CON+FO group, n=15) and diabetes mellitus + FO group (DM+FO group, n=15) were fed a standard containing 4% FO (1.5 g/kg/d, 34% EPA, 24% DHA, Sheng Tianyu Biotechnology, China). The chemical structure of EPA and DHA are shown in Figure 6. Seventy-two hours after STZ administration, rats with a blood glucose level more than 16.7mmol were identified as DM models. A timeline of the test procedures is shown in Figure 7.

### Morris water maze test

Ten weeks after FO treatment, the learning and memory abilities of the rats were assessed using the MWM [27]. On day 89, all the animals were subjected to trainings trials that lasted 4 days. Rats were trained to find hidden platform submerged 2 cm below the water surface. If the rats failed to find the platform in 60 s, they were gently placed onto the platform and allowed to stay for 10 s. The time spent finding and climbing onto the platform was recorded as the escape latency. On day 93, the hidden platform was removed, and a probe trial test was conducted to evaluate the spatial memory of the rats, which were given 60 s to swim freely in the pool. The time the rats spent in the target quadrant, the number of times the rats crossed the platform, and the swimming speed was recorded.

### Histopathological examinations

After the MWM test, morphological alterations in the cortex and hippocampus (CA1) were analyzed by hematoxylin and eosin (H&E). Rats were anesthetized with sodium pentobarbital (60 mg/kg) and the head was cut off to remove the brain tissue in an ice tray. The brain tissue was fixed by perfusing 200–300 ml of 4% paraformaldehyde. The tissue was collected, postfixed, and embedded in paraffin. The paraffin tissue blocks were cut into 5-mm-thick sagittal sections for H&E staining. Cortex and hippocampal cells were counted by light microscopy.

### Quantitative real-time PCR analysis

The transcription levels of IL-1β, IL-6 and TNF-α, IL-4 and IL-10 were measured using quantitative real-time PCR. Cortex and hippocampal tissues were prepared to extract total RNA using RNA-Solv Reagent (Omega). Reverse transcription was conducted with 2 μg of RNA using ReverTra Ace (Toyobo) and Oligo(dT)18(TaKaRa). qRT-PCR was performed on SYBR Premix Ex Taq (TaKaRa) using a ViiA 7 RealTime PCR System (Thermo). All procedures were performed following the manufacturer’s instructions. The primers were shown as the following: IL-1β fwd 5′-AGGTCGTCATCATCCCACGAG-3′, rev 5′-GCTGTGGCAGCTACCTATGTCTTG-3′; IL-6 fwd 5′-CACTCCGGAGAGGAGACAAG-3′, rev 5′-ACAGTGCATCATCGCTGTTC-3′; TNF-α fwd 5′-GAGAGATTGGCTGCTGGAAC-3′, rev 5′-TGGAGACCATGATGACCGTA-3′; IL-4 fwd 5′-CTCAGGACGGTAAGTGTCCCTG-3′, rev 5′-GTTCAGGCGCGCATGTTGTCG-3′; IL-10 fwd 5′-GGCTTCCTTACTCCGCGTTGG-3′, rev 5′-TCCTAGAACTGGAACCACGGCT-3′; β-actin fwd 5′-CAGCCGCGAGATTACCTACAA-3′, rev 5′-CTTTGCATGCCTCCGCTCCGT-3′.

### Measurement of ROS and oxidative stress

Tissue from the cortex and hippocampus were homogenized and prepared for cytokine assays. The levels of ROS and MDA, and the activities of CAT, SOD and GSH were evaluated using ROS assay kit, lipid peroxidation (MDA) assay kit, CAT assay kit, SOD assay kit and GSH assay kit (Nanjing Jiancheng Bioengineering Institute, China), respectively. All the procedures followed the manufacturer’s instructions.

### Western blot analysis

Total protein was extracted from cortex and hippocampus tissues. BCA kit was used to determine the concentration of the total protein. Equal quantities of protein (20 mg/lane) were loaded on a 10% SDS-PAGE gel and transferred to a PVDF membrane (200 mA, 2 h). The membranes were blocked for 2h in 5% skim milk at 37°C, and then incubated at 4°C overnight with primary antibodies, against Nrf2 (1:1000, ab31163, Abcam, USA), HO-1(1:500, ab13243, Abcam, USA) and β-actin (1:4000, Proteintech, USA). The membranes were incubated with secondary antibodies for 40 min. The bands were analyzed using Image J software.

### Data analysis

Data analysis was conducted using SPSS 25.0 (IBM software) and Sigma Plot10 (Sigma Plot software). The data are shown as the means ± SEM. Bonferroni means separation test was performed after ANOVA. Results were only reported when significant differences (*P*<0.05, *P*<0.01 or *P*<0.001) were observed.

### Ethics statement

The rat experiments were approved by the Animal Ethics Welfare Committee of North China University of Science and Technology.
